# Mechanisms of PARP inhibitor resistance in cancer and insights into the DNA damage response

**DOI:** 10.1186/s13073-018-0612-8

**Published:** 2018-12-28

**Authors:** Paola Francica, Sven Rottenberg

**Affiliations:** 1 Institute of Animal Pathology, Vetsuisse Faculty, Länggassstrasse, University of Bern, 3012 Bern, Switzerland; 2 Division of Molecular Pathology, The Netherlands Cancer Institute, 1006 BE Amsterdam, The Netherlands

## Abstract

Inhibitors of poly(ADP-ribose) polymerase (PARPi) have entered the clinic for the treatment of patients with cancers that lack homology-directed DNA repair, but drug resistance remains a clinical hurdle. Recent advances in the identification of PARPi resistance mechanisms have yielded a better understanding of DNA end protection and the relevance of endogenous poly(ADP-ribose) glycohydrolase, highlighting new vulnerabilities.

## How do PARP inhibitors work?

In 2005, two landmark studies demonstrated the striking sensitivity of BRCA1- and BRCA2-deficient tumor cells to poly(ADP-ribose) polymerase (PARP) inhibition, and since then several PARP inhibitors (PARPi) have been developed for clinical use (reviewed in [1]). As a prime example of the concept of synthetic lethality in cancer, PARPi have scored successes in the treatment of patients with *BRCA1/2*-mutated cancers [1]. BRCA1 and BRCA2 are key players in the error-free repair of DNA double-strand breaks (DSBs) by homologous recombination (HR). When cells become HR-deficient because of the loss of BRCA1 or BRCA2, DSBs need to be repaired by alternative error-prone repair pathways, resulting in chromosome deletions, translocations, and subsequent cell death. This vulnerability is exploited by treating HR-deficient tumors with PARPi.

The molecular mechanisms that underlie the selective killing of HR-deficient cells by PARPi are not yet completely clear. Initially, it was thought that PARPi cause an increase in DNA single-strand breaks (SSBs). When encountered by a replication fork, these breaks result in toxic DSBs in BRCA1/2-defective cells. This model was challenged by the discovery that PARP can be trapped on DNA at the sites of unrepaired SSBs (reviewed in [1]) and that this causes the lethal effect of PARPi. Yet the exact nature of the DNA structures on which PARP enzymes are trapped remains undefined. Recently, Hanzlikova et al. [2] suggested that unligated Okazaki fragments (short DNA sequences that are synthesized discontinuously to create the lagging strand during DNA replication) resulting from PARPi are the responsible structures. The unligated fragments may require HR-mediated repair for their removal, either directly as single-strand gaps or following their conversion into DSBs by nucleases or DNA replication fork collapse. Hanzlikova et al. [2] concluded that PARP1 also acts as a sensor of unligated Okazaki fragments during DNA replication, facilitating their repair. In addition, Massimo Lopes and colleagues suggested that PARPi treatment promotes premature, RECQ1-dependent restart of reversed replication forks. This results in unrestrained replication fork progression and in the subsequent accumulation of DSBs (reviewed in [1]).

Surprisingly, PARPi may also prevent tumorigenesis by impeding the interactions of PARP1 with the cyclic GMP–AMP synthase (cGAS) [3]. The cytosolic DNA sensor cGAS has recently been shown to link genomic instability to the innate immune response. DNA damage-induced nuclear translocation of cGAS inhibits HR by interacting directly with PARP1 and thereby suppressing the PARP1-timeless complex [3]. It is unlikely though that the effect of PARPi on cGAS compensates for the tumorigenic potential of PARP1 trapped on chromatin.

## Learning from mechanisms of PARPi resistance

As with all targeted therapies that have entered the clinic, the benefit of PARPi in patients with *BRCA1/2*-mutated tumors is counteracted by the emergence of drug resistance (reviewed in [1]). Understanding the underlying mechanisms may not only be useful for attempts to counteract PARPi resistance; this knowledge has also yielded novel insights into basic mechanisms of the DNA damage response. Among the resistance mechanisms identified to date, (partial) restoration of homology-directed DNA repair is frequently observed in various model systems and in patients, highlighting the HR defect as the Achilles heel for PARPi (reviewed in [1]).

An obvious mechanism of HR restoration is the reactivation of BRCA1/2 function as a result of secondary genetic alterations (reviewed in [1]). More intriguing are mechanisms of BRCA1-independent partial HR restoration: first, this type of HR restoration was shown to occur owing to inactivation of the p53-binding protein 1 (53BP1) (reviewed in [1]). 53BP1 plays a crucial role in maintaining the balance between HR and non-homologous end joining (NHEJ), which is shifted toward NHEJ in BRCA1-deficient cells. Mechanistically, 53BP1 promotes NHEJ by inhibiting the extensive nucleolytic resection of DNA termini required for HR repair. Hence, loss of 53BP1 function facilitates BRCA1-independent end resection and conveys PARPi resistance. Follow-up studies identified that the inactivation of downstream factors of 53BP1-mediated repair, such as RIF1 and REV7, also results in the restoration of DNA end resection and thereby promotes homology-mediated repair (reviewed in [1]). However, the ultimate effectors of the 53BP1 pathway responsible for DNA end protection remain unknown.

Recently, several groups have identified the molecular mechanisms by which 53BP1 mediates its function in DNA repair (reviewed in [1]). Using ascorbate peroxidase-based proximity labeling or functional genetic screens for PARPi resistance factors in BRCA1-deficient cells, a new 53BP1 effector complex called shieldin was discovered. This complex comprises C20orf196 (also known as SHLD1), FAM35A (SHLD2), CTC-534A2.2 (SHLD3) and REV7. Shieldin functions as a downstream effector in the 53BP1 pathway by restraining DNA end resection. Mechanistically, the shieldin complex localizes directly to DSB sites and its loss impairs NHEJ, leads to defective immunoglobulin class switching, and causes hyper-resection. Mutations in genes that encode the shieldin subunits cause PARPi resistance in BRCA1-deficient cells but not in BRCA2-deficient cells. Ghezraoui et al. [4] found that shieldin is involved in distinct DSB repair activities of the 53BP1 pathway: it is essential for DNA end protection and NHEJ during class-switch recombination, but it is dispensable for REV7-dependent interstrand cross-link repair.

Another factor that regulates 53BP1-dependent NHEJ is DYNLL1 [5]. Binding of DYNLL1 to 53BP1 stimulates its recruitment to DSB sites, and stabilizes its interaction with DNA damage-associated chromatin. Moreover, He et al. [6] found that DYNLL1 also binds to MRE11 to limit DNA end resection in BRCA1-deficient cells. Given its role in the degradation of reversed replication forks, it would be interesting to investigate whether MRE11 inhibition by DYNLL1 binding also protects replication forks, similar to the loss of PTIP (reviewed in [1]).

Moreover, we and others found that PARPi resistance in BRCA1-deficient cells is caused by the loss of the CTC1–STN1–TEN1 (CST) complex, suggesting that CST–Polα-mediated fill-in helps to control the repair of DSBs by the 53BP1-RIF1-REV7-Shieldin pathway [7, 8]. Although it remains to be established whether CST-mediated inhibition of end resection at non-telomeric DSBs is dependent on Polα, the CST complex might contribute to preventing resection at DSBs in addition to its role in telomere maintenance.

In contrast to these mechanisms of partial HR restoration in BRCA1-deficient cells, HR-independent resistance to PARPi has been enigmatic. However, Gogola et al. [9] recently made an interesting observation. They combined genetic screens with multi-omics analysis of matched PARPi-sensitive and -resistant *Brca2*-mutated mouse mammary tumors and observed that loss of PAR glycohydrolase (PARG), the main enzyme responsible for degrading nuclear PAR, was involved in a major resistance mechanism [9]. Our data show that endogenous PARG activity is crucial for the success of PARPi therapy and that PARG suppression restores PARP1 signaling upon PARPi treatment. Hence, PARG activity may be another useful predictive marker for PARPi therapy.

Intriguingly, HR restoration was not observed in BRCA2-deficient tumor cells that acquired PARPi resistance ([9] and unpublished). These data raise the question of whether BRCA1 is less essential than BRCA2 for homology-directed DNA repair. To date, our data show that loss of the 53BP1-RIF1-REV7-Shieldin-CST pathway only partially restores BRCA1 deficiency. It remains to be shown whether loss of members of this pathway can be fully compensated for in mice with a complete *Brca1* depletion.

## Implications for translation into the clinic

Despite the plethora of PARPi resistance mechanisms, there is also hope: the analysis of PARPi resistance mechanisms revealed new vulnerabilities that can be exploited therapeutically. For instance, we and others have shown that loss of the 53BP1-RIF1-REV7-Shieldin-CST pathway in PARPi-resistant BRCA1-deficient cells results in hypersensitivity to ionizing radiation [10, 11]. This is most probably due to the role of this pathway in NHEJ: in contrast to PARPi, DSB induction by ionizing radiation is less dependent on the S phase of the cell cycle and therefore relies more on repair through the NHEJ pathway than on HR. We also found increased radiosensitivity of PARPi-resistant tumors that lost PARG [9]. This may be caused by the depletion of the pool of non-PARylated PARP1 necessary to catalyze DNA repair. Radiotherapy or a treatment with radiomimetic drugs might therefore serve as a useful treatment option for PARPi-resistant tumors in which no genetic reversion of BRCA1/2 is detected. It also raises the question of whether alternating treatment cycles of PARPi and radiomimetic drugs would be more successful than the PARPi maintenance treatment currently used in platinum-sensitive ovarian cancer.

## Abbreviations

cGAS: Cyclic GMP–AMP synthase
CST: CTC1–STN1–TEN1
DSB: DNA double-strand break
HR: Homologous recombination
NHEJ: Non-homologous end joining
PARG: PAR glycohydrolase
PARP: Poly(ADP-ribose) polymerase
PARPi: PARP inhibitor
SSB: DNA single-strand break
